# Association between screen time and suspected developmental coordination disorder in preschoolers: A national population-based study in China

**DOI:** 10.3389/fpubh.2023.1152321

**Published:** 2023-03-27

**Authors:** Shanshan Geng, Weijie Wang, Liping Huang, Jinhong Xie, Gareth J. Williams, Charlie Baker, Wenchong Du, Jing Hua

**Affiliations:** ^1^The Women’s and Children’s Health Care Department of Shanghai First Maternity and Infant Hospital, Tongji University School of Medicine, Shanghai, China; ^2^School of Social Sciences, Nottingham Trent University, Nottingham, United Kingdom; ^3^Department of Psychology, Nottingham Trent University, Nottingham, United Kingdom; ^4^Shanghai Key Laboratory of Maternal Fetal Medicine, Shanghai First Maternity and Infant Hospital, School of Medicine, Tongji University, Shanghai, China

**Keywords:** excessive screen exposure, screen time before sleep, suspected DCD, one-child family, preschoolers

## Abstract

**Introduction:**

Excessive screen exposure (ESE) is a growing global public health concern. This study aims to investigate the potential association between ESE and suspected developmental coordination disorder (DCD) in Chinese pre-schoolers, with or without siblings.

**Method:**

A retrospective cohort study was conducted, involving 126,433 children from 551 cities in China. The Little Developmental Coordination Disorder Questionnaire (LDCDQ) was employed to evaluate motor impairment in children, while parents provided information on their children’s screen time in the past year. A mixed and multi-level logistic regression model was used to analyze the associations of all screen exposure measurements from the past year with LDCDQ scores and the risk of suspected DCD.

**Results:**

The prevalence of excessive screen exposure was 67.6% (>1 h per day) and 28.9% (>2 h per day) in Chinese pre-schoolers. One hour’s increase in weekday daily screen time, weekend daily screen time, and screen time before sleep in the past year was associated with a decreased total score of the LDCDQ (β were −0.690, −0.398, and −1.587, *p* < 0.001) and an increased risk of suspected DCD by 15.3%, 9.1%, and 46.8% when adjusting for the child, family and maternal health characteristics. Excessive screen exposure decreased the total LDCDQ scores by 1.335 (>1 vs. ≤1 h) and 1.162 (>2 vs. ≤2 h) and increased risks of suspected DCD by 44.0% (>1 vs. ≤1 h) and 31.1% (>2 vs. ≤2 h) with statistical significance (each *p* < 0.05). The stratified analysis showed that the association between screen time and LDCDQ score was stronger in children without siblings than in those with siblings.

**Conclusion:**

The risk of suspected DCD was highest for screen time exposure before bed compared with average weekday and weekend exposures. Parents should be advised to prevent their children from using electronic screens unsupervised, especially in one-child families.

## Introduction

Evidence suggests that excessive screen exposure (ESE) time in early childhood is associated with child development and health (1–4). The COVID-19 pandemic has triggered a marked increase in sedentary behavior, notably excessive screen time among children, with potential long-term implications for their developmental outcomes (5). Several studies had documented that screen time in young children can be longer than 2 h per day at 30 months old in the United Kingdom (6), the United States (7), and India (8), and children at 18 and 30 months old were reported to watch more than 4 h of TV per day in a Japanese study (9). Reports also suggested that the screen exposure time varied from 21% to 98% in middle-income countries, and 10% to 93.7% in high-income countries, respectively (10).

Screen exposure time normally includes time spent watching TV, or using a smartphone, a computer, or a tablet. According to the previous literature, two standards were commonly used to define ESE, and daily screen time exceeding 1 h (9, 11–13) or 2 h (14–17) per day is generally considered excessive. ESE has been linked to delayed development of language (11, 18), negative psychosocial development, and cognitive and socioemotional development (19). Research has indicated that preschool children who engage in screen time exceeding 2 h per day display increased emotion dysregulation, diminished prosocial behavior (20, 21), elevated inattention, and hyperactivity (22, 23). Moreover, recent studies have increasingly reported associations between ESE and developmental disorders, such as Autism Spectrum Disorder (ASD) and Attention-Deficit/Hyperactivity Disorder (24–26). However, no study has examined the effects of screen time and ESE on developmental coordination disorder (DCD), a neurodevelopmental disorder which affects child motor and coordination function and occurs in 5%–6% of children (27).

In the present study, we conducted a national retrospective cohort study in preschoolers aged 3–5 years old in China, aiming to investigate the association between screen exposure time during weekdays, weekends, and before sleep in the past year with motor development and the risk of DCD. We also explored the effects of excessive screen exposure time on DCD using two cut-off standards of screen exposure (>1 h per day) (9, 11–13, 28) and >2 h per day (14–17). Additionally, the role of sibling presence in the association between screen time and suspected DCD was also examined.

## Methods

### Study design and population

Data on motor development was extracted from the Chinese National Cohort of Motor Development (CNCMD) (29). Stratified cluster sampling was used to ensure that the study participants were representative of the Chinese population. The China 2018–2019 National Census was used for stratification by geographic region, age, sex, and socioeconomic status (SES). Nurseries were invited to participate in the study. Class teachers at the nurseries which agreed to participate distributed notifications to parents to complete an online questionnaire, and researchers’ contact details were provided so parents or teachers could make contact if they had queries about the study. An online questionnaire system was used in the study for data collection.

**Figure 1:**
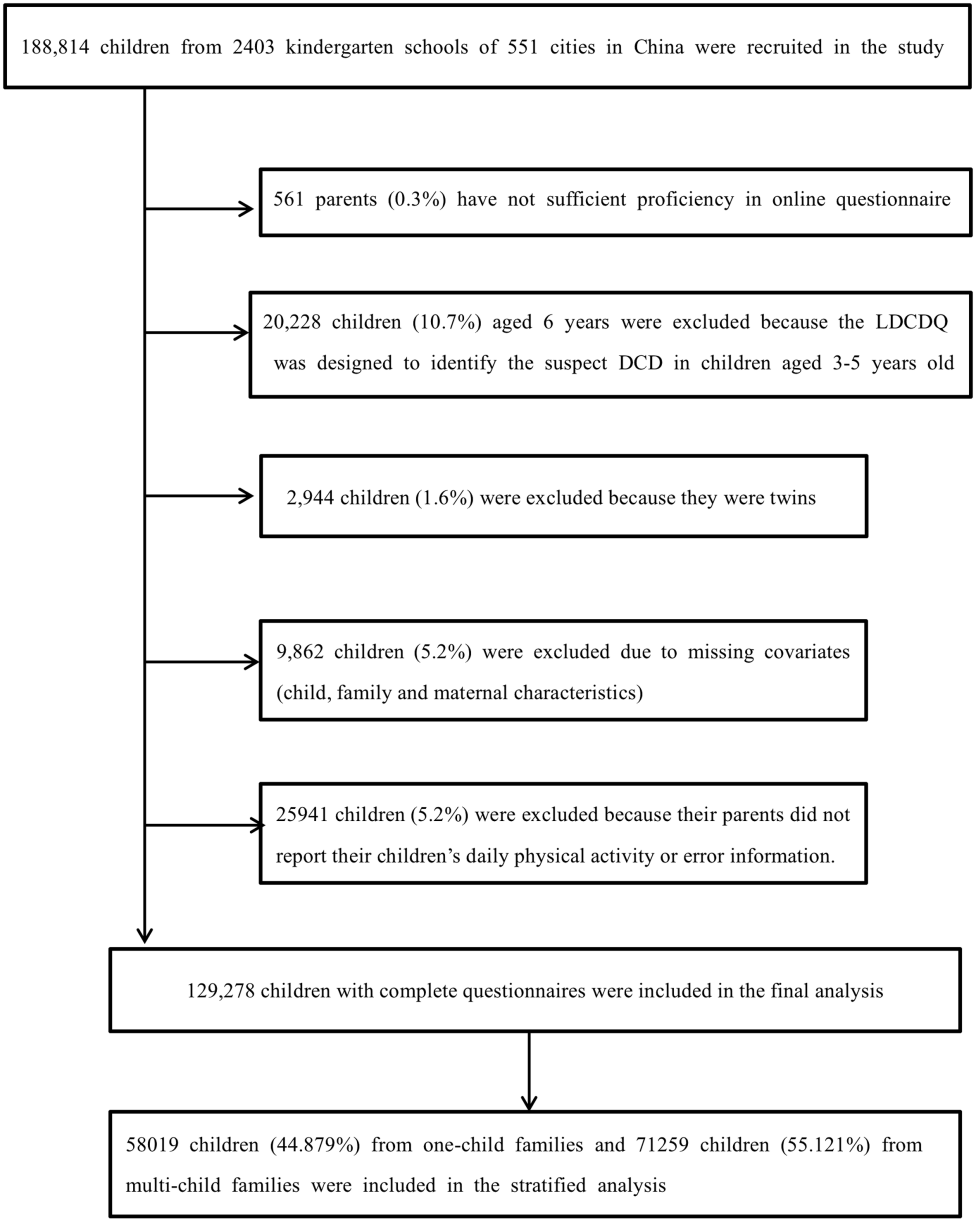
Flowchart of the study population.

Data were collected from 1st April 2018 to 3rd December 2019, and 188,814 pre-schoolers from 2,403 mainstream nurseries in 551 cities of China were recruited in total. A total of 129,278 children were included in the analysis of the current study (Figure 1). Informed consent has been obtained from all participants. The study was approved by the ethics committee of Shanghai First Maternity and Infant Hospital (KS18156).

### Exposure

Following the same measurement used in previous studies (1–4), parents were asked to provide the average time in a day in the past year their child spent watching TV, using a smartphone or computer or other tablets with a screen by answering three questions: (1). Consider the typical situation in the past whole year, how many minutes on a weekday does your child usually spend watching TV, using a smartphone, a computer, or a tablet? (2). Consider the typical situation in the past whole year, how many minutes on a weekend day does your child usually spend watching TV, using a smartphone, a computer, or a tablet? and (3). Consider the typical situation in the past whole year, how many minutes does your child usually spend watching TV, using a smartphone, a computer, or a tablet before sleep? ***Weekday daily screen time***, ***weekend screen time***, and ***screen time before sleep*** was also converted into hours. According to the previous literature (30, 31), the average daily time in hours a child spent in screen exposure (*daily screen time*) was weighted with 5/7 given to weekday screen time and 2/7 given to weekend screen time. Additionally, ***ESE*** was defined as ***daily screen time*** exceeding one (9, 11–13) and two (14–17) hours per day.

### Outcomes

The motor performance of children was assessed using the Little Developmental Coordination Disorder Questionnaire (LDCDQ), which was developed as a screening tool for motor coordination difficulties in 3- and 4-year-old children (32) and can also be extended for use with children up to 5 years (33). The LDCDQ consists of 15 questions divided into three sub-categories: control during movement/gross motor, fine motor skills, and general coordination. Each category contains five items; for each item, parents are asked to compare the performance of their child with that of children of the same age and sex and to rate their performance on a 5-point Likert scale, with 1 = not at all relevant to my child and 5 = extremely relevant to my child. Each sub-category has a maximum score of 25. Scores are summed to give a maximum total score of 75, with higher scores indicating a higher level of motor proficiency. The LDCDQ has been demonstrated to be a valid and reliable measurement in Chinese children (34). Per previous recommendations (33, 35), we used the age- and sex-specific norms of the LDCDQ and cut-off scores based on a national sample in China to indicate suspected impairments of motor coordination (“suspected DCD” with LDCDQ ≤ 15 percentile; “probably not DCD” with LDCDQ > 15 percentile).

### Other covariates

A wide range of child personal, family, and maternal health characteristics as potential confounders (which might be related to both screen exposure and DCD), and adjust for in the analysis (Table A1). BMI is computed by children’s height and weight [BMI = weight (kg)/height(m)] (36). Family structures were grouped into three categories: single-parent family, nuclear family, and families with more than two generations in the same household. Maternal age were grouped into three categories: “<30,” “30–34,” and “>34” years (37). Other Maternal complications were defined according to the International Classification of Diseases-Revision 10 (ICD-10), i.e., if the mother had one of the following maternal complications: vaginal bleeding during pregnancy, risk of miscarriage, use of antibiotics, use of fertility drugs, intrauterine distress, or fetal asphyxia. The daily physical activity was divided into two categories by different time duration: low level (<180 min) and high level (≥180 min) according to the National Association of Sport and Physical Education (NASPE) guidelines of the United States for preschool-aged children (aged 3–5 years).

### Statistical analysis

A mixed model utilizing a random intercept (we hypothesized that there was no interaction between nursery types and the total LDCDQ scores) was used to investigate the associations of screen time with the total score and sub-scores of LDCDQ. A multi-level logistic regression model was used to determine the strength of association for different gestational ages associated with poor motor performance (“suspected DCD” was defined as LDCDQ ≤ 15th percentile; “probably not DCD” was defined as LDCDQ > 15th percentile). The nurseries as primary sampling units and other potential confounders (child personal, family, and maternal health characteristics as described above) were all considered in these models. All covariates as mentioned earlier were controlled for in the analysis. In stratified analysis, the above associations of screen time with suspected DCD in one-child and multi-child families were compared using the Z test. Analyses were carried out using LMER, GLMER procedures using R version 4.0.1. and *p* < 0.05 was denoted as statistically significant.

## Results

### Characteristics of the participants

Of the 129,278 children included in the final analysis, the weekday daily screen time, the weekend daily screen time, and the screen time before sleep were 1.275, 2.584, and 0.515 h, with a standard deviation of 0.948, 1.847, and 0.506, respectively. A total of 86,728 children (67.1%) had more than 1 h of daily screen time, and 37,362 children (28.9%) had more than 2 h of daily screen exposure. The mean of the total score of LDCDQ, and the sub-scores in motor control, writing/fine motor, and general coordination were 67.817, 22.907, 22.716, and 22.194, with a standard deviation of 8.927, 3.102, 3.154, and 3.218, respectively. Per total LDCDQ scores, 19,969 children (15.447%) were defined as suspected DCD and 109,309 children (84.553%) were defined as probably not DCD The child, family characteristics and maternal health during pregnancy in the study population were shown in Table 1.

**Table 1:** The child, family characteristics and maternal health during pregnancy in the study population (*n* = 129,278).

	Total	Excessive screen exposure (>1 h)		Excessive screen exposure (>2 h)	Suspected DCD	
		Yes	No	Yes	Yes	No
**Child characteristics**						
Children’s age (M, SD)	3.950 (0.789)	3.960 (0.789)	3.927 (0.787)	3.955 (0.788)	3.941 (0.781)	3.951 (0.790)
BMI (M, SD)	15.602 (1.617)	15.606 (1.612)	15.588 (1.631)	15.705 (1.683)	15.617 (1.637)	15.599 (1.613)
**Gender (*n*%)**						
Male	67,780 (52.430)	51,888 (52.200)	15,892 (53.193)	20,567 (55.048)	10,107 (50.613)	57,673 (52.761)
Female	61,498 (47.570)	47,514 (47.800)	13,984 (46.807)	16,795 (44.952)	9,862 (49.387)	51,636 (47.239)
**Physical activities**						
≥180 min	65,008 (50.285)	44,842 (51.704)	20,166 (47.394)	20,796 (55.661)	9,405 (47.098)	55,603 (50.868)
<180 min	64,270 (49.715)	41,886 (48.296)	22,384 (52.606)	16,566 (44.339)	10,564 (52.902)	53,706 (49.132)
**Right handedness (*n*%)**						
No	7,962 (6.224)	6,359 (6.473)	1,603 (5.402)	2,384 (6.381)	1,449 (7.393)	6,513 (6.013)
Yes	119,960 (93.776)	91,887 (93.527)	28,073 (94.598)	34,978 (93.619)	18,150 (92.607)	101,810 (93.987)
**Eyesight (*n*%)**						
Normal	116,546 (94.958)	88,917 (94.773)	27,629 (95.559)	35,881 (96.036)	17,481 (94.512)	99,065 (95.037)
Abnormal	6,188 (5.042)	4,904 (5.227)	1,284 (4.441)	1,481 (3.964)	1,015 (5.488)	5,173 (4.963)
**Low birth weight (*n*%)**						
No	124,385 (96.215)	95,614 (96.189)	28,771 (96.301)	35,901 (96.090)	19,007 (95.183)	105,378 (96.404)
Yes	4,893 (3.785)	3,788 (3.811)	1,105 (3.699)	1,461 (3.910)	962 (4.817)	3,931 (3.596)
**Preterm birth (*n*%)**						
No	103,344 (79.939)	79,034 (79.509)	24,310 (81.370)	29,270 (78.342)	15,231 (76.273)	88,113 (80.609)
Yes	25,934 (20.061)	20,368 (20.491)	5,566 (18.630)	8,092 (21.658)	4,738 (23.727)	21,196 (19.391)
**Delivery mode**						
Vaginal delivery	67,160 (51.950)	52,019 (52.332)	15,141 (50.679)	19,323 (51.718)	10,483 (52.496)	56,677 (51.850)
Delivery with cesarean section	62,118 (48.050)	47,383 (47.668)	14,735 (49.321)	18,039 (48.282)	9,486 (47.504)	52,632 (48.150)
**NICU admission**						
No	115,926 (89.672)	88,833 (89.367)	27,093 (90.685)	33,325 (89.195)	17,686 (88.567)	98,240 (89.874)
Yes	13,352 (10.328)	10,569 (10.633)	2,783 (9.315)	4,037 (10.805)	2,283 (11.433)	11,069 (10.126)
**Other developmental disorders**						
No	128,302 (99.245)	98,605 (99.198)	29,697 (99.401)	37,037 (99.130)	19,617 (98.237)	108,685 (99.429)
Yes	976 (0.755)	797 (0.802)	179 (0.599)	325 (0.870)	352 (1.763)	624 (0.571)
**Psychiatric medication**						
No	128,242 (99.199)	98,542 (99.135)	29,700 (99.411)	37,046 (99.154)	19,776 (99.034)	108,466 (99.229)
Yes	1,036 (0.801)	860 (0.865)	176 (0.589)	316 (0.846)	193 (0.966)	843 (0.771)
**Family characteristics**						
**Higher education of mother (*n*%)**						
No	58,862 (45.531)	45,756 (46.031)	13,106 (43.868)	22,294 (59.670)	12,929 (64.745)	45,933 (42.021)
Yes	70,416 (54.469)	53,646 (53.969)	16,770 (56.132)	15,068 (40.330)	7,040 (35.255)	63,376 (57.979)
**Higher education of father (*n*%)**						
No	60,013 (46.422)	46,684 (46.965)	13,329 (44.614)	22,574 (60.420)	12,702 (63.609)	47,311 (43.282)
Yes	69,265 (53.578)	52,718 (53.035)	16,547 (55.386)	14,788 (39.580)	7,267 (36.391)	61,998 (56.718)
**Mother’s occupation (*n*%)**						
Employed	107,606 (83.236)	82,711 (83.209)	24,895 (83.328)	30,164 (80.734)	15,625 (78.246)	91,981 (84.148)
Unemployed	21,672 (16.764)	16,691 (16.791)	4,981 (16.672)	7,198 (19.266)	4,344 (21.754)	17,328 (15.852)
**Father’s occupation (*n*%)**						
Employed	125,431 (97.024)	96,348 (96.928)	29,083 (97.346)	35,992 (96.333)	18,986 (95.077)	109,445 (97.380)
Unemployed	3,847 (2.976)	3,054 (3.072)	793 (2.654)	1,370 (3.667)	983 (4.923)	2,864 (2.620)
**Family annual per-capita income (RMB)** ^**a**^ **(*n*%)**						
Below	32,851 (25.411)	25,566 (25.720)	7,285 (24.384)	10,395 (27.822)	6,168 (30.888)	26,683 (24.411)
Above or equal to	96,427 (74.589)	73,836 (74.280)	22,591 (75.616)	26,967 (72.178)	13,801 (69.112)	82,626 (75.589)
**Family structure (*n*%)**						
Single families	3,200 (2.475)	2,534 (2.549)	666 (2.229)	1,164 (3.115)	632 (3.165)	2,568 (2.349)
Nuclear families	79,952 (61.845)	60,764 (61.130)	19,188 (64.225)	22,782 (60.976)	13,071 (65.456)	66,881 (61.185)
Extended families	46,126 (35.680)	36,104 (36.321)	10,022 (33.545)	13,416 (35.908)	6,266 (32.379)	39,860 (36.465)
**The number of children in the family (*n*%)**						
One	58,019 (44.879)	43,542 (43.804)	14,477 (48.457)	18,440 (49.355)	9,700 (48.575)	48,319 (44.204)
Two or more	71,259 (55.121)	55,860 (56.196)	15,399 (51.543)	18,922 (50.645)	10,269 (51.425)	60,990 (55.796)
**Maternal health during pregnancy**						
**Maternal age at delivery (*n*%)**						
<30	95,915 (74.193)	74,577 (75.026)	21,338 (71.422)	28,748 (76.944)	14,922 (74.726)	80,993 (74.095)
30–34	25,007 (19.344)	18,729 (18.842)	6,278 (21.014)	6,326 (16.932)	3,609 (18.073)	21,398 (19.576)
≥35	8,356 (6.464)	6,096 (6.133)	2,260 (7.565)	2,288 (6.124)	1,438 (7.201)	6,918 (6.329)
**Smoking or passive smoking during pregnancy (*n*%)**						
No	93,487 (72.315)	70,349 (70.772)	23,138 (77.447)	24,713 (66.145)	13,925 (69.733)	79,562 (72.786)
Yes	35,791 (27.685)	29,053 (29.228)	6,738 (22.553)	12,649 (33.855)	6,044 (30.267)	29,747 (27.214)
**Maternal complications during pregnancy** ^**b**^						
No (n%)	123,032 (95.169)	94,378 (94.946)	28,654 (95.910)	35,592 (95.263)	19,041 (95.353)	103,991 (95.135)
Yes	6,246 (4.831)	5,024 (5.054)	1,222 (4.090)	1770 (4.737)	928 (4.647)	5,318 (4.865)

a The national average family per-capita income of the year before the survey time.

b Having one of the following maternal complications during pregnancy including gestational diabetes, hypertensive disorders, vaginal bleeding during pregnancy, at risk of miscarriage, use of antibiotics, use of fertility drugs, intrauterine distress, fetal asphyxia.

### Associations of screen time and ESE with the LDCDQ scores

The results showed that 1 h’s increase in weekday daily screen time, weekend daily screen time, and screen time before sleep was associated with a decreased total score of the LDCDQ (β were −0.690, −0.398, and −1.587, *p* < 0.001) when adjusting for physical activity and family, maternal and child’s characteristics. One hour’s increase in weekday daily screen time, weekend daily screen time, and screen time before sleep was associated with all three sub-scores of the LDCDQ: the sub-score of motor control (adjusted β = −0.179, −0.101, and −0.480, each *p* < 0.001), writing/fine motor (adjusted β = −0.265, −0.151, and −0.583, each *p* < 0.001), and general coordination (adjusted β = −0.246, −0.147, and −0.526, each *p* < 0.001) when adjusting for all the covariates.

ESE in the past year was also associated with a decreased total score of the LDCDQ, sub-score of motor control, writing/fine motor, and general coordination (>1 h: adjusted β = −1.335, −0.345, −0.534, and −0.457; >2 h: adjusted β = −1.162, −0.278, −0.453. and −0.432, each *p* < 0.001). The crude and adjusted β and 95% CI were shown in Table 2.

**Table 2:** The association between screen time and score of the LDCDQ in preschoolers (*n* = 129,278).

Screen exposure	Total score		Motor control		Writing/fine motor		General coordination	
	Crude β (95% CI)	Adjusted β^a^ (95% CI)	Crude β (95% CI)	Adjusted β^a^ (95% CI)	Crude β (95% CI)	Adjusted β^a^ (95% CI)	Crude β (95% CI)	Adjusted β^a^ (95% CI)
**Total (*n* = 129,278)**								
Screen time during weekday (hours)	−0.950^***^	−0.690^***^	−0.250^***^	−0.179^***^	−0.356^***^	−0.265^***^	−0.347^***^	−0.246^***^
	(−1.001, −0.898)	(−0.741, −0.638)	(−0.268, −0.232)	(−0.197, −0.161)	(−0.374, −0.338)	(−0.283, −0.247)	(−0.365, −0.328)	(−0.264, −0.227)
Screen time during weekend (hours)	−0.514^***^	−0.398^***^	−0.133^***^	−0.101^***^	−0.187^***^	−0.151^***^	−0.196^***^	−0.147^***^
	(−0.541, −0.487)	(−0.425, −0.372)	(−0.143, −0.124)	(−0.110, −0.091)	(−0.196, −0.177)	(−0.160, −0.142)	(−0.205, −0.186)	(−0.157, −0.138)
Screen time before sleep (hours)	−1.992^***^	−1.587^***^	−0.599^***^	−0.480^***^	−0.716^***^	−0.583^***^	−0.682^***^	−0.526^***^
	(−2.082, −1.901)	(−1.677, −1.498)	(−0.631, −0.568)	(−0.511, −0.448)	(−0.748, −0.684)	(−0.614, −0.551)	(−0.715, −0.650)	(−0.558, −0.494)
Excessive screen exposure > 1 vs. ≤1 h	−1.807^***^	−1.335^***^	−0.482^***^	−0.345^***^	−0.685^***^	−0.534^***^	−0.647^***^	−0.457^***^
	(−1.912, −1.703)	(−1.439, −1.232)	(−0.518, −0.446)	(−0.382, −0.309)	(−0.722, −0.648)	(−0.571, −0.498)	(−0.685, −0.610)	(−0.494, −0.419)
>2 vs. ≤2 h	−1.587^***^	−1.162^***^	−0.393^***^	−0.278^***^	−0.597^***^	−0.453^***^	−0.604^***^	−0.432^***^
	(−1.695, −1.480)	(−1.269, −1.056)	(−0.430, −0.355)	(−0.315, −0.240)	(−0.635, −0.559)	(−0.491, −0.416)	(−0.643, −0.565)	(−0.471, −0.394)
**One-child family (*n* = 58,019)**								
Screen time during weekday (hours)	−1.053^#^^***^	−0.770^#^^***^	−0.294^#^^***^	−0.213^#^^***^	−0.378^***^	−0.282^***^	−0.385^***^	−0.276^***^
	(−1.131, −0.975)	(−0.847, −0.692)	(−0.322, −0.267)	(−0.241, −0.186)	(−0.405, −0.350)	(−0.309, −0.254)	(−0.413, −0.357)	(−0.303, −0.248)
Screen time during weekend (hours)	−0.545^***^	−0.415^***^	−0.147^***^	−0.109^***^	−0.193^***^	−0.153^***^	−0.206^***^	−0.153^***^
	(−0.584, −0.505)	(−0.454, −0.375)	(−0.161, −0.133)	(−0.123, −0.095)	(−0.207, −0.179)	(−0.167, −0.139)	(−0.220, −0.192)	(−0.167, −0.139)
Screen time before sleep (hours)	−2.172^#^^***^	−1.734^#^^***^	−0.672^#^***	−0.538^***^	−0.770^***^	−0.627^***^	−0.737^***^	−0.570^***^
	(−2.307,−2.036)	(−1.868, −1.600)	(−0.719, −0.625)	(−0.585, −0.491)	(−0.818, −0.723)	(−0.674, −0.581)	(−0.785, −0.688)	(−0.619, −0.522)
Excessive screen exposure > 1 vs. ≤1 h	−2.102^***^	−1.517^***^	−0.597^#^^***^	−0.422^#^^***^	−0.764^***^	−0.576^***^	−0.750^***^	−0.521^***^
	(−2.268, −1.935)	(−1.682, −0.352)	(−0.655, −0.540)	(−0.479, −0.364)	(−0.822,−0.705)	(−0.634, −0.519)	(−0.810, −0.690)	(−0.580, −0.461)
>2 vs. ≤2 h	−1.693^#^^***^	−1.226^#^^***^	−0.430^#^^***^	−0.298^#^^***^	−0.616^***^	−0.459^***^	−0.655^***^	−0.470^***^
	(−1.854, −1.531)	(−1.386, −1.067)	(−0.486, −0.373)	(−0.354, −0.242)	(−0.672,-0.559)	(−0.515, −0.403)	(−0.713, −0.597)	(−0.527, −0.413)
**Multi-child family (*n* = 71,259)**								
Screen time during weekday (hours)	−0.912^***^	−0.629^***^	−0.227^***^	−0.150^***^	−0.356^***^	−0.257^***^	−0.333^***^	−0.223^***^
	(−0.980, −0.843)	(−0.697, −0.561)	(−0.251, −0.203)	(−0.174, −0.126)	(−0.380, −0.332)	(−0.281, −0.233)	(−0.358, −0.308)	(−0.247, −0.198)
Screen time during weekend (hours)	−0.516^***^	−0.390^***^	−0.130^***^	−0.094^***^	−0.191^***^	−0.152^***^	−0.198^***^	−0.144^***^
	(−0.552, −0.480)	(−0.425, −0.354)	(−0.142, −0.117)	(−0.107, −0.081)	(−0.204, −0.179)	(−0.165, −0.139)	(−0.211, −0.185)	(−0.157, −0.131)
Screen time before sleep (hours)	−1.925^***^	−1.480^***^	−0.564^***^	−0.435^***^	−0.700^***^	−0.552^***^	−0.688^***^	−0.494^***^
	(−2.047, −1.803)	(−1.601, −1.360)	(−0.606, −0.521)	(−0.477, −0.392)	(−0.744, −0.657)	(−0.594, −0.510)	(−0.712, −0.624)	(−0.538, −0.451)
Excessive screen exposure > 1 vs. ≤1 h	−1.691^***^	−1.228^***^	−0.427^***^	−0.295^***^	−0.664^***^	−0.514^***^	−0.608^***^	−0.419^***^
	(−1.824, −1.557)	(−1.360, −1.096)	(−0.474, −0.380)	(−0.342, −0.249)	(−0.711, −0.617)	(−0.561, −0.468)	(−0.656, −0.560)	(−0.467, −0.372)
>2 vs. ≤2 h	−1.593^***^	−1.128^***^	−0.388^***^	−0.263^***^	−0.617^***^	−0.469^***^	−0.595^***^	−0.407^***^
	(−1.737, −1.448)	(−1.271, −0.986)	(−0.439, −0.338)	(−0.313, −0.212)	(−0.668, −0.556)	(−0.509, −0.409)	(−0.648, −0.543)	(−0.459, −0.355)

a Adjusting for child and family characteristics and maternal health during pregnancy.

# The associations between screen time and LDCDQ scores were different in one-child family from those of multi-child family with statistically significance.

*** *p* < 0.001.

### Associations of screen time and ESE with suspected DCD

One hour’s increase in weekday daily screen time, weekend daily screen time, and screen time before sleep in the past year was associated with an increased risk of suspected DCD by 15.3%, 9.1%, and 46.8% when adjusting for physical activity and family, maternal and child’s characteristics. ESE increased the risk of suspected DCD by 44.0% (>1 vs. ≤1 h) and 31.1% (>2 vs. ≤2 h) when adjusting for the same covariates. The crude and adjusted OR and 95%CI were shown in Table 3.

**Table 3:** The association between screen time and risk of DCD in preschoolers (*n* = 129,278).

Screen exposure	Suspected DCD		Crude OR(95% CI)	Adjusted OR^a^ (95% CI)
	Yes	No		
Screen time during weekday (hours) M (SD)	1.475 (1.044)	1.238 (0.925)	1.208^***^	1.153^***^
			(1.190, 1.226)	(1.135, 1.171)
Screen time during weekend (hours) M (SD)	3.020 (1.994)	2.505 (1.808)	1.120^***^	1.091^***^
			(1.112, 1.129)	(1.082, 1.099)
Screen time before sleep (hours) M (SD)	0.662 (0.603)	0.489 (0.519)	1.618^***^	1.468^***^
			(1.575, 1.662)	(1.428, 1.509)
**Excessive screen exposure (*n*%)**				
≤1 h	4,576 (22.916)	37,974 (34.74)	Ref	Ref
>1 h	15,393 (77.084)	71,335 (65.260)	1.629^***^	1.440^***^
			(1.571, 1.689)	(1.387, 1.494)
**Excessive screen exposure (*n*%)**				
≤2 h	12,569 (62.943)	79,347 (72.590)	Ref	Ref
>2 h	7,400 (37.057)	29,962 (27.410)	1.433^***^	1.311^***^
			(1.387, 1.481)	(1.268, 1.355)

a Adjusting for child and family characteristics and maternal health during pregnancy.

*** *p* < 0.001.

### Stratified analysis by one-child and two-child family

**Figure 2:**
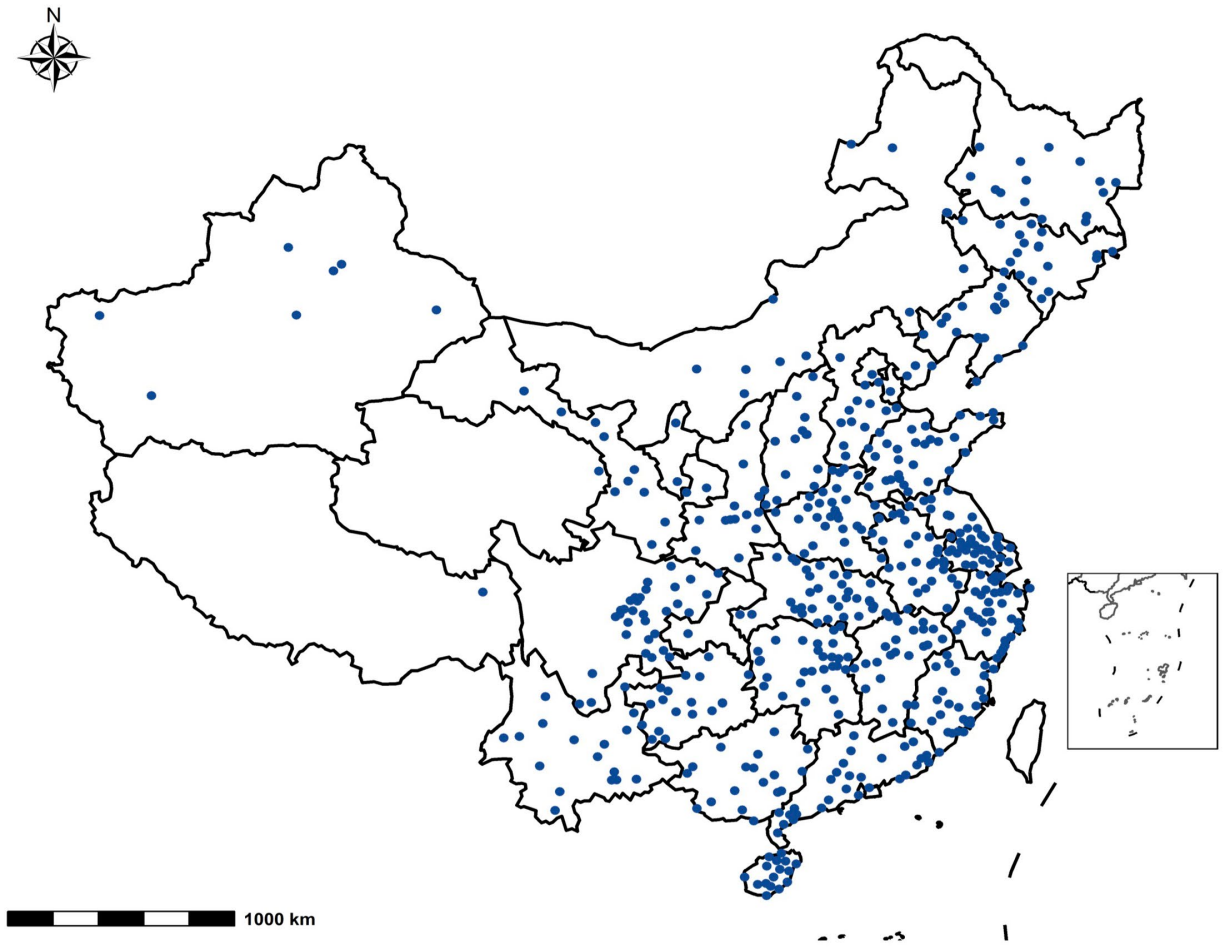
Distribution of 551 participant cities from 31 provinces/municipalities/autonomous regions in mainland China.

**Figure 3:**
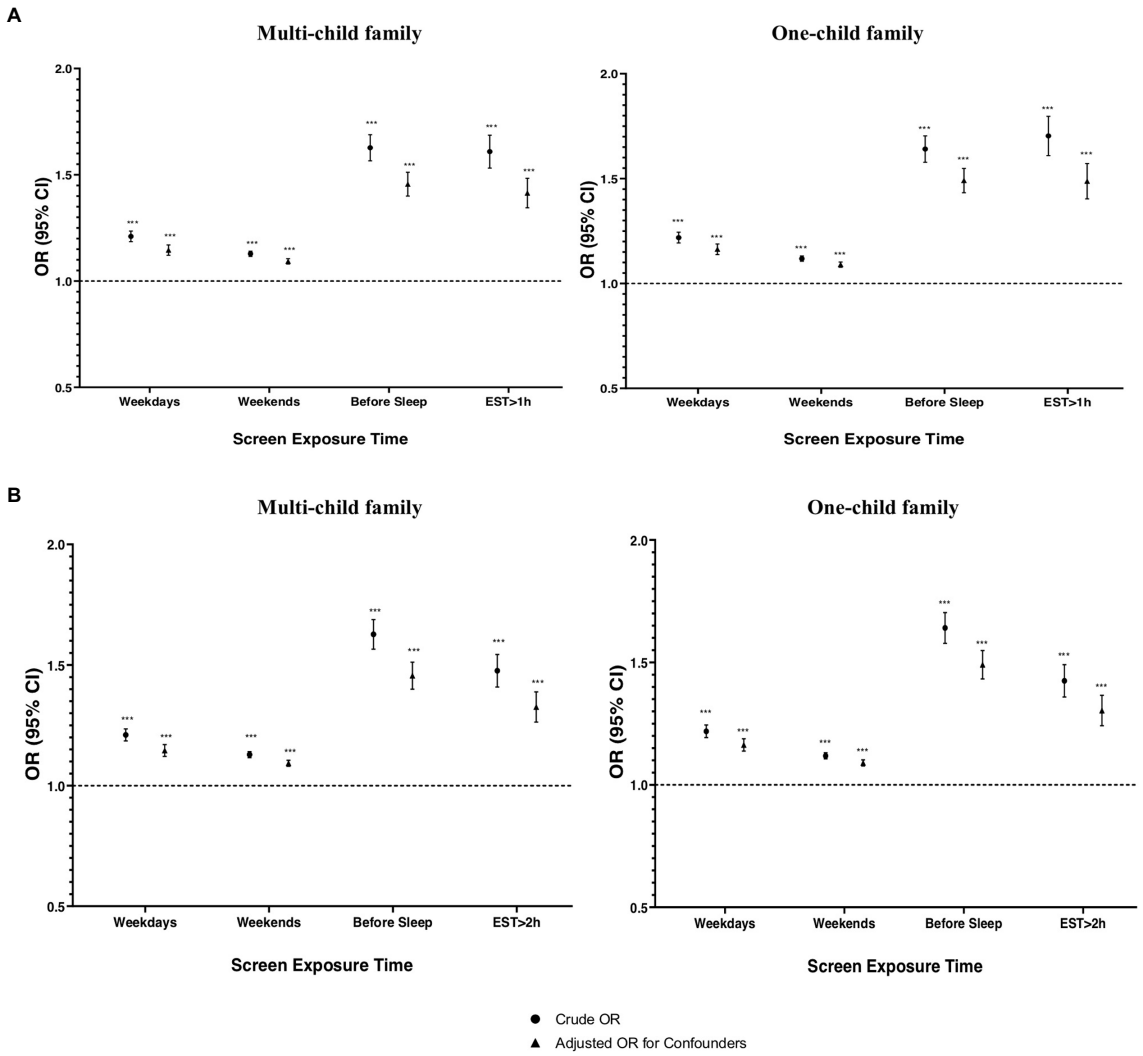
The association between screen time and suspected DCD in multi child and one-child family when adjusting or not adjusting for child and family characteristics and maternal health during pregnancy (*n* = 129,278). **(A)** Excessive screen exposure is defined as more than 1 h per day. **(B)** Excessive screen exposure is defined as more than 2 h per day.

The stratified analysis showed that the statistically significant associations of screen time with the LDCDQ scores and suspected DCD remained in both one-child and multi-child families (Figures 2, 3). However, the association between prolonged screen time (weekday, before sleep) and the LDCDQ scores (total score, motor control, and general coordination) in one-child families were stronger than those in multi-child families with statistical significance (each *p* < 0.05).

### Sensitivity analyses

The associations of screen time and ESE with the total score of LDCDQ (Figure A1) and risk of suspected DCD (Figure A2) remained robust before and after adjusting for the covariates. The strengths of these results did not change significantly when adjusting for the covariates, respectively (Figures A1, A2).

## Discussion

The present study examined the association between screen time and DCD, using a large nationally representative sample. There were significant associations between all measurements of screen exposure time in the past year with motor impairment measurements and the risk of DCD. The strongest association was found for screen exposure time before sleep when compared to weekday daily screen time and weekend daily screen time. Additionally, the association between screen exposure time and the risk of DCD was stronger in children without siblings than in those with siblings.

We found that longer weekday and weekend daily screen time in the past year was associated with negatively affected motor performance including gross motor, fine motor, and balance when adjusting for a wide range of confounding factors including physical activities. Previous studies have reported associations between screen time and fundamental motor skills (38, 39), and associations between prolonged screen exposure time and both decreased physical activity engagement and fine and gross motor performances. Additionally, screen exposure time has been reported to negatively relate to attention-related patterns generated by (EEG) in preschool children (40). While complicated and distinct, there are specific relationships between motor performance and cognitive processes (41), due to the close connections between brain development and motor abilities (42), which are consequential for early childhood development. With neuroscientific evidence having linked ESE to the delayed development of cognitive processes (43, 44), we can infer that ESE may also affect motor development.

Our results also suggested an association between prolonged screen exposure time on weekdays and weekends in the past year and increased risk of DCD. Increased screen exposure time has previously been associated with lower microstructural integrity of brain white matter tracts in preschool-aged children (45). Children with DCD also showed significant brain differences in motor and sensorimotor white matter pathways when compared with controls (46, 47). Furthermore, evidence suggested that higher screen exposure is independently associated with lower serum Brain-derived neurotrophic factor (BDNF) levels (48). The BDNF genotype regulates both the inhibitory and excitatory circuits in the human primary motor cortex which mostly relate to motor controls (49). Therefore, ESE might lead to a higher risk of motor impairments through the change in the microstructural integrity of the brain. It should be noted that the association we found between ESE and the risk of DCD was stronger using the WHO recommendation (>1 h per day) compared to exceeding 2 h per day. Exceeding 1 h of screen time in a day increased the vulnerability in physical health and well-being, social competence, and communication skills among other domains of developmental health (50). Using 1 h as the excessive screen exposure daily cut-off standard showed a higher prediction power compared to 2 h. Our study provides new evidence to support that one-hour maximum screen time per day is an optimal recommendation for pre-schoolers.

### The strongest association was found for screen time before sleep

One of our important findings is that the screen time before sleep had the strongest association between the LDCDQ score and the risk of suspected DCD, compared to weekday and weekend daily screen time. One possible explanation is the effect of screen exposure on melatonin. Exposure to blue light from backlit electronic screens has been found to inhibit melatonin production (51). Melatonin has been found to improve motor coordination in ethanol-hungover mice (52), and treatment with melatonin could promote motor performance in nocturnal animals (53). Additionally, prolonged screen exposure was associated with later bedtimes and shorter sleep duration in pre-schoolers (4), which can also lead to circadian discrepancy (54) and decreased motor performance during the day (55).

### The moderating effect of siblings

With the relaxation of China’s one-child policy in 2016, there has been sustained interest in the role of siblings in a multi-child dynamic on child development. A stronger association between prolonged screen time and motor competence was found in one-child families in the current study compared to multi-child families. Previous studies found that being the only child in a family is a risk factor for DCD and motor development delay (56, 57), and suggested the positive influence of the presence of a sibling on motor development. Studies suggest that older siblings can provide good role models that younger children can imitate (58), which can then, therefore, help to decrease the time needed by parents to teach basic motor skills to the younger ones. The presence of siblings in the family context is especially influential for motor development after 24 months of age because siblings provide cooperative activities such as play and challenges that improve cognitive, social, emotional, and physical development (59), which may also moderate the negative influence of screen exposure.

## Strengths and limitations

There are several strengths to this study, one is the size and cross-sectoral nature of the sample. This was the first study based on a large nationally representative sample and is also one of the few that examined the sibling effect of screen exposure on a neurodevelopmental disorder. Limitations of the current study included that self-reported information on childhood adversities may produce a differential recall bias and result in an inaccurate estimation of total and direct effects. However, the majority of parents do not have awareness of motor impairment or DCD (60) and children with DCD are rarely diagnosed in China (61). Therefore, the two groups with parents of children with and without suspected DCD in the current study were less likely to have different recalling accuracy when providing the information of their children, and misclassification of the two groups is unlikely to be different. Therefore, the possibility of differential recall bias can be considered minimal in the current study. Moreover, it is often insufficient to control for confounding factors in a retrospective study. However, we included a wide range of confounders to adjust for in the analysis in the current study, although some potential confounders such as the presence of another child in the family with a physical or neurodevelopmental delay could also be controlled for because it could also affect the motor development of children. It should also be noticed that although we asked the parents to recall the general screen exposure time on a typical day in the past year, the current study was a retrospective cohort study, and our research results cannot support any causal relationship among variables. Additionally, we did not use a validated scale to assess screen exposure in our study. Further research with a longitudinal design using a scaled measurement might be needed to explain the mechanisms linking screen exposure and DCD.

## Conclusion

Digital devices have been used more widely by young children to receive information, and parents should be advised to prevent their children from using the screen excessively which can affect their normal neurodevelopment. Limiting screen time exposure could form an integral part of child healthcare, which can be achieved by the combined participation of parents, guardians, and healthcare professionals. Future studies should focus on effective practices to reduce screen time in children.

## Data availability statement

The raw data supporting the conclusions of this article will be made available by the authors, without undue reservation.

## Ethics statement

The study was approved by the Ethics Committee of Shanghai First Maternity and Infant Hospital (KS18156). Written informed consent to participate in this study was provided by the participants’ legal guardian/next of kin.

## Author contributions

WD and JH: integrity of the data, accuracy of the data analysis, and concept and design. SG and JH: drafting of the manuscript. SG, WW, LH, and JX: acquisition, analysis, and interpretation of data. JH, WD, GW, and CB: critical revision of the manuscript for important intellectual content. SG, JH, and WD: administrative, technical, or material support and obtained funding. All authors contributed to the article and approved the submitted version.

## Funding

This study was supported by the National Natural Science Foundation of China (81673179), the Science and Technology Commission of Shanghai Municipality (21DZ2202000 and 19140903100), Shanghai Municipal Health Commission (2020YJZX0213), and Shanghai Pudong New Area Health Commission (PW2020D-11).

## Conflict of interest

The authors declare that the research was conducted in the absence of any commercial or financial relationships that could be construed as a potential conflict of interest.

## Publisher’s note

All claims expressed in this article are solely those of the authors and do not necessarily represent those of their affiliated organizations, or those of the publisher, the editors and the reviewers. Any product that may be evaluated in this article, or claim that may be made by its manufacturer, is not guaranteed or endorsed by the publisher.
