# Supplementary material for: Association between screen time and suspected developmental coordination disorder in preschoolers: A national population-based study in China
Source: Front Public Health. 2023 Mar 27;11:1152321. doi: 10.3389/fpubh.2023.1152321 (PMC10083417; doi:10.3389/fpubh.2023.1152321)
Supplement: Supplementary file 1 [file Data_Sheet_1.docx]

**TABLE A1 The definition of covariates which have been controlled in the model of our study**

| Name of factors | Definitions |
| --- | --- |
| ***Family characteristics*** |  |
| Higher education of mother/father | Higher education refer to tertiary education leading to award of an academic degree. |
| Mother/father’s occupation | We divided the occupation into ‘employed’ and ‘unemployed’. People with jobs are employed. People who are jobless, looking for a job, and available for work are unemployed. |
| Family annual per-capita income | The national average family per-capita income of the year before the survey time |
| Family structure | Family structures were classified into three types: three-generation (or more) family, nuclear family, and single-parent family. The ‘three-generation (or more) family’ refers to the child living with their parents and grandparents, which is a traditional family structure in China; “nuclear family” refers to the child living only with their parents, and “single mother or father” means the child lives with one of their parents. |
| The number of children in the family | Number of biological or adoptive children in the family. |
|  |  |
| ***Maternal health during pregnancy*** |  |
| Maternal age at delivery | We divided maternal age into three age bands: ‘<30’, ‘30-34’ and ‘>34’ years old consistent with previous literature. |
| Smoking or passive smoking during pregnancy | Smoking refer to the mother had been ‘active’ smoking during pregnancy. And passive smoking defined as inhalation of [tobacco smoke](https://en.wikipedia.org/wiki/Tobacco_smoke) in a environment(anyone smoke inside home or in indoor areas where mother working during pregnancy). |
| Maternal complications during pregnancy | Having one of the following maternal complications during pregnancy including gestational diabetes, hypertensive disorders, vaginal bleeding during pregnancy, at risk of miscarriage, use of antibiotics, use of fertility drugs, intrauterine distress, fetal asphyxia according to the International Statistical Classification of Diseases and Related Health Problems, Tenth Revision. |
| Delivery Mode | A vaginal delivery is the birth of babies in humans through the vagina. Delivery with caesarean section is the surgical delivery of a baby through a cut (incision) made in the mother's abdomen and uterus. |
|  |  |
| ***Child characteristics*** |  |
| Children’s age | The children’s age were computed by ‘the date of investigation minus the date of birth’ |
| Gender | Male or female |
| Right handedness | Using the right hand habitually or more easily than the left |
| Low birth weight | Low birth weight has been defined by WHO as weight at birth of < 2500 grams. |
| Preterm birth | Preterm birth has been defined by WHO as any birth before 37 completed weeks of gestation. |
| NICU admission | NICU admission refer to a newborn who has a health condition that needs special care(eg. Respiratory Distress Syndrome, Prematurity, Sepsis or infection, Hypoglycemia, Perinatal depression, Maternal chorioamnionitis). |
| Eyesight | The normal eyesight refer to a reference value above which visual acuity is considered normal is called 6/6 vision, the USA equivalent of which is 20/20 vision. |
| BMI | An indicator of obesity which is based on height and weight (BMI=weight(kg)/height(m)) according to the WHO BMI classification. |
| Other developmental disorders | Other developmental disorders included autism spectrum disorder, attention-deficit/hyperactivity disorder, learning disorders, etc. |
| Psychiatric medication | A psychiatric medication is a psychoactive drug taken to exert an effect on the chemical makeup of the brain and nervous system, which might influence the mind, emotions, and behavior of children. |
|  |  |

#

**FIGURE A1. Sensitivity analysis for screen exposure time and the total score of LDCDQ**

^a^ Adjusting for child characteristics

^b^ Adjusting for family characteristics

^c^Adjusting for maternal health during pregnancy

^d^Adjusting for all above confounders

**FIGURE A2. Sensitivity analysis for screen exposure time and the risk of suspected DCD**

^a^ Adjusting for child characteristics

^b^ Adjusting for family characteristics

^c^Adjusting for maternal health during pregnancy

^d^Adjusting for all above confounders
